# Dendritic cells matured with recombinant human sperm associated antigen 9 (rhSPAG9) induce CD4^+^, CD8^+^ T cells and activate NK cells: a potential candidate molecule for immunotherapy in cervical cancer

**DOI:** 10.1186/s12935-021-01951-7

**Published:** 2021-09-07

**Authors:** Hemavathi Dhandapani, Hascitha Jayakumar, Abirami Seetharaman, Shirley Sunder Singh, Selvaluxmy Ganeshrajah, Nirmala Jagadish, Anil Suri, Rajkumar Thangarajan, Priya Ramanathan

**Affiliations:** 1 Department of Molecular Oncology, Cancer Institute (WIA), Dr. Krishnamurthi Campus, 38, SardarPatel Road, Chennai, 600036 India; 2 Department of Radiation Oncology, Cancer Institute (WIA), Dr. Krishnamurthi Campus, 38, SardarPatel Road, Chennai, 600036 India; 3 Department of Pathology, Cancer Institute (WIA), Dr. Krishnamurthi Campus, 38, SardarPatel Road, Chennai, 600036 India; 4 Cancer Microarray, Genes and Proteins Laboratory, National Institute of Immunology, Aruna Asaf Ali Marg, New Delhi, 110067 India

**Keywords:** SPAG9, Cisplatin, Th1, IFNγ, Immunotherapy, Dendritic cells

## Abstract

**Background:**

Dendritic cell (DC)-based immunotherapy is capable of activating the immune system and in particular tumor-specific cytotoxic T lymphocytes (CTLs) to eradicate the tumor. However, major limitations are the availability of autologous tumor cells as antigenic source and the selection of antigen that may have potential to activate both CD4^+^ and CD8^+^ T cells in immune-specific manner. Recently, we reported the expression of sperm associated antigen 9 (SPAG9) that is associated with various types of malignancies including cervical cancer. We examined the recombinant human SPAG9 (rhSPAG9) as an antigenic source for generating efficient DCs to stimulate CD4^+^ and CD8^+^ T cell responses for future DCs-based vaccine trials in cervical cancer patients.

**Methods:**

Human monocytes derived DCs were pulsed with different concentrations (250 ng/ml to 1000 ng/ml) of recombinant human SPAG9 (rhSPAG9) and evaluated for their phenotypic and functional ability. The efficacy of DCs primed with 750 ng/ml of rhSPAG9 (SPDCs) was compared with DCs primed with autologous tumor lysates (TLDCs), to induce CD4^+^, CD8^+^ T cells and activating NK cells. In addition, we investigated the effect of the chemotherapeutic drug cisplatin on phenotypic and functional potential of SPDCs.

**Results:**

Phenotypic and functional characterization of DCs pulsed with 750 ng/ml rhSPAG9 was found to be optimal and effective for priming DCs. SPDCs were also capable of stimulating allogeneic T cells similar to TLDCs. SPDCs showed a statistically insignificant increase in the expression of maturation marker CD83 and migration towards CCL19 and CCL21 compared with TLDCs (CD83; *P* = 0.4; migration; *P* = 0.2). In contrast, although TLDCs showed better proliferation and secretion of Th1 cytokines (IL12p40, IL12p70 and IFNγ) compared to SPDCs, this difference was not statistically significant (IL12p40, *P* = 0.06). Further we also observed that clinical dose of cisplatin (200 µM) treated SPDCs were able to stimulate the proliferation of cytotoxic T lymphocytes without increasing the FOXP3^+^ Tregs in autologous co-cultures.

**Conclusions:**

In summary, in order to overcome the limitation of the availability of autologous tumor cells as antigenic sources, our present strategy provides an insight to consider rhSPAG9 as a strong immunogen for DC-based immunotherapy for cervical cancer trials and warrants further studies. This is the first report to suggest that rhSPAG9 is an effective antigen for pulsing DCs that are capable of eliciting a potent Th1 response which, in turn, may help in decreasing the tumor burden when used along with a cisplatin based combinatorial regimen for therapeutic intervention.

**Supplementary Information:**

The online version contains supplementary material available at 10.1186/s12935-021-01951-7.

## Background

Cervical cancer is the leading cause of cancer related death among women worldwide and continues to be a major public health problem affecting middle-aged women, particularly in less-resourced countries [1]. Cervical cancer’s cure rate is 90% in early stage disease. However, these rates drop to 50% or less in the advanced stages (IIIb and above) particularly in India [2]. Hence novel therapeutic strategies that are effective in reducing the tumor burden, are still needed desperately. Accordingly, we focused our efforts on developing a therapeutic vaccine for patients presenting with advanced stages of disease, which may be combined with existing concurrent chemo-radiotherapy regimens to improve cure rates.

Dendritic cells have emerged as important cell based therapeutic adjuvants due to their ability to cross present antigens to the host immune system and express ample co-stimulatory molecules. However, availability of whole tumor antigens has posed the biggest challenge to DC vaccine production. Hence the use of cancer testis antigen (CTA), due to their unique expression, have made them attractive peptide vaccine candidates, particularly MAGE-A and NY-ESO1 which have been used as therapeutic entities in several clinical trials [3–6]. Recent studies showed that restricted NY-ESO-1 immunogenic peptides in combination with various adjuvants exhibited potent anti-tumor response [7, 8]. However, two other phase II trials with a single MAGEA3 peptide did not show an improvement in disease free survival [9, 10]. In contrast to peptides or peptide mixtures, whole protein CT antigens may be suitable for peptide processing and presentation across several HLA types when used for priming autologous DCs [11, 12].

Recently, sperm associated antigen 9 (SPAG9) has been shown to be expressed in various cancer types such as epithelial ovarian cancer (EOC-90%) [13], renal cell carcinoma (RCC-88%) [14], colorectal cancer (74%) [15], breast cancer (88%) [16] and cervical cancer (82%) [17]. Studies on SPAG9 also showed that it is associated with cellular proliferation, migration and invasion of cancer cells [18–21], and is capable of eliciting humoral immune responses in a majority of epithelial ovarian cancers (67%) [13], breast cancers (80%) [16], cervical cancers (80%) [17], renal cell carcinoma (77%) [14], colorectal cancer (74%) [15] and hepatocellular carcinoma [22]. However its antigenicity in invoking a cell mediated immune response has not been studied until now.

Combinatorial regimens utilizing chemotherapy and immunotherapy may decrease the tumor burden as well as the immunosuppressive cells in the tumor microenvironment facilitating pronounced synergistic effects. Several clinical trials conducted in solid tumors also showed an increased overall survival of patients when cell based immunotherapy with DC vaccines was combined with chemotherapy regimens [23, 24]. In evidence, our own phase I clinical trial [25] employed whole tumor lysate as antigen for priming DCs followed by cisplatin chemotherapy which showed regression of metastatic lung lesion as well as clearance of disease in the primary site in one patient who continues to be disease free for the past eleven years. In line with our observation, Spanos et al. showed that cisplatin treatment at 20 mg/m^2^ in HPV-positive immune competent mice caused subjective clearance of tumor while the same was not attained in immune-incompetent mice which indicate the important role of immune cells in abrogating HPV-positive tumor cells [26]. Hence, evaluating the efficacy of dendritic cells and DCs stimulated T cells in the presence of chemotherapeutic agents such as cisplatin may be necessary to account for any difference in such functionality linked to specific antigens affecting their antigenicity. This is the first study to assess the potential role of SPAG9 protein as an antigenic source for dendritic cell priming. First, we primed immature DCs with increasing dosage of rhSPAG9 and determined the optimal priming dose. The efficacy of rhSPAG9 primed DCs (SPDCs) was examined using immunophenotyping and functional assays using allogenic responders. As our earlier studies in-vitro [27] and our phase I trial in cervical cancer patients [25], has demonstrated the efficacy of autologous tumor lysate pulsed DCs (TLDCs), as a next step after optimizing the rhSPAG9 dose for priming, TLDCs were generated from the same patient and used to compare to assess the functional characteristics of SPDCs against autologous responders. Finally, the ability of SPDCs to elicit a Th1 response in combination with cisplatin at various concentrations was also assessed. Our present study has laid down the foundation for undertaking Phase II cervical cancer trials employing rhSPAG9 protein as an antigenic source for DC based vaccines.

## Methods

### Patient samples

Blood samples (20 ml) were collected from 12 patients (P1–P12) who were diagnosed with cervical cancer at Cancer Institute, Adyar, Chennai for isolation of PBMCs and DCs culture. Tumor punch biopsy samples were collected from 4 patients (P6–P9) to prepare tumor lysates for the generation of TLDCs. For all further in vitro studies, DCs and PBMCs were also obtained (DCs from all P1–P12 patients and PBMCs from P7–P12 patients for autologous and allogenic studies). In addition, 20 ml blood samples were also obtained from eight healthy donors (N1–N8) for isolation of PBMCs using Ficoll (GE Healthcare, USA) for allogenic studies. The monocytes were depleted by plastic adhesion subsequently to enrich for lymphocytes and used for allogeneic studies. The study was approved by the Institutional ethical committee from Cancer Institute Adyar, Chennai. The duly signed consent forms were obtained from each patient and healthy donor prior to the study. Tumor tissues were collected for tumor lysate in Hank’s balanced salt solution (HBSS) containing 100 IU of penicillin, streptomycin and gentamycin and in 10% formalin for immunohistochemistry.

### Immunohistochemistry for SPAG9 expression

The SPAG9 protein expression was examined in cervical cancer tissue sections by IHC as described earlier [17]. Briefly, the cervical cancer tissue sections (4 μm) were deparaffinizedand subsequently rehydrated using different gradients of alcohol. Polyclonal antibodies to recombinant SPAG9 were generated and purified as described earlier [13]. Tissue sections were probed with anti-SPAG9 antibody or control IgGat 1:100 dilution overnight at 4 °C in humid chamber. After washing thrice with phosphate buffer saline (PBS)-0.05% Tween 20, the tissue sections were incubated with horseradish peroxidase-conjugated goat anti-rat IgG (Jackson ImmunoResearch Laboratories, West Grove, PA) and visualized using DAKO Envision kit (K500711-2) following the manufacturer’s instructions and counterstained with hematoxylin and mounted with 1,3-diethyl-8-phenylxanthine [(DPX), Sigma-Aldrich, St. Louis, MO]. The images of tissue sections were captured using Nikon microscope (Nikon Instrument lnc., NY, USA).

### Antibodies and flow cytometry

For phenotypic analysis of dendritic cells: Fluorochrome conjugated antibodies CD14-PC5, CD80-FITC, HLADR-ECD, CD40-PE and CD86-PE conjugated were purchased from Beckman Coulter Inc (Carlsbad, CA, USA). The antibody against maturation marker CD83-conjugated to APC was purchased from Bio Legend (San Diego, CA, USA).

For phenotyping proliferating PBMCs, anti-CD56-PE, anti-CD4-PC5 anti-CD8-APC, anti- CD25-ECD and anti-CD3-PC7 antibodies were purchased from Beckman Coulter Inc (Carlsbad, CA, USA). Anti-FOXP3-PE was purchased from BioLegendInc (San Diego, CA, USA). Flow cytometry was performed as described previously [27]. All the samples were acquired using MoFlo XDP flow cytometer configured with three different lasers-blue (488 nm), violet (405 nm) red (640 nm) analyzed using Summit 5.2 software (both Beckman Coulter Inc, Carlsbad, CA, USA).

### DCs generation and priming

Unprimed and TLDCs were generated from peripheral blood mononuclear cells (PBMCs) isolated using a FicollPaque Plus (GE Healthcare, UK) density gradient. Monocytes were enriched by plastic adherence for DCs differentiation and were either primed with tumor lysates (TLDCs) prepared from single cell suspensions of punch biopsy samples or matured directly (unprimed DCs) as described earlier [27]. SPDCs were generated using GMP grade rhSPAG9 protein [28] trademark SPAGNII™ (GMP grade rhSPAG9 was manufactured by Syngene International Pvt. Ltd, India; SPAGNII™). The purified rhSPAG9 protein was used at different concentrations of 250 ng, 500 ng, 750 ng and 1000 ng/ml for priming and generation of SPDCs following same protocol used for the generation of TLDCs.

### Wash out (WO) test

The mature dendritic cells were washed and suspended in serum free CellGro DC medium (Cell Genix, Freiburg, Germany) for 24 h. Adherence and changes in morphology following cytokine withdrawal were monitored microscopically and counts were obtained by trypan blue exclusion. The washout (WO) test medium was stored in protein low bind tubes (Eppendorf, Hamburg, Germany) at − 70 °C.

### Phenotypic characterization of mature DCs

For immunophenotypic analysis, 5 × 10^4^ mature DCs were incubated for 10 min with 5% FBS and then stained with anti- CD14-PC5, CD40-PE, HLADR-ECD, CD86-PE, CD80-FITC and anti-CD83-APC for 20 min at RT. Cells were also stained with appropriate isotypic controls and washed twice with PBS, fixed and suspended in 2% PFA (Sigma Aldrich, St. Louis, MO, USA) until analysis.

### Proliferation assay with rhSPAG9 primed DCs

For the proliferation assay, non adherent cells enriched for lymphocytes after monocyte depletion for DCs culture were prepared from allogenic and autologous PBMCs. Matured DCs primed with rhSPAG9 (250, 500, 750 and 1000 ng/ml per million cells), were co-cultured with PBMCs, stained with carboxyfluorescein, succinimidyl ester [CFSE (Invitrogen, Carlsbad, CA, USA)]. Briefly, SPDCs were co-cultured at the ratio of 1:50 (DCs: allogeneic PBMCs) and 1:10 (DCs: autologous PBMCs) to check for proliferation as described in our previous study [27]. The cells were cultured for 8 days. The cells were centrifuged at 1580 rpm and subsequently pellet was resuspended and washed with PBS, blocked with 5% FBS. The cells were further probed with anti-CD4-PC5, anti-CD8-APC, anti-CD56-PE, anti-CD25-ECD, anti-FOXP3-PE antibodies and Propidium iodide (1 µg/ml; Himedia, India) to verify the response of the autologous or allogeneic PBMCs upon stimulation with SPDCs. Unstimulated PBMCs were used as negative controls in all experiments and the percentage proliferation of these cells has been subtracted from all DC stimulated wells to arrive at the representative data. Cells were acquired using a MoFlo XDP cell sorter/ Flow cytometer (Beckman Coulter, Carlsbad, CA, USA) and FCS express 7 was used for analyzing the proliferating population.

### Migration assay

Migratory capacity of TLDCs and SPDCs was assessed in a 24 well plate using (5µ) transwell inserts (Corning, Corning, NY, USA). The lower chamber of each well was filled with serum free medium containing 300 ng/ml CCL19 and 250 ng/ml CCL21 (R&D systems, Minneapolis, MN, USA) and the DCs were incubated for 3 h at 37 °C. Subsequently, the cells were centrifuged and then re-suspended in total volume of 300 µl PBS**.** Samples were analyzed in a flow cytometer using a fixed flow rate. The number of cells acquired per minute through flow cytometer was calculated as described previously [27].

### IFNγ analysis by ELISPOT assay

ELISPOT assay was done to measure antigen-specific IFNγ release by PBMCs removed after monocytes attachment. All steps were carried out as per manufacturer’s protocol [CTLTechnologies, Shaker Heights, OH, USA]. The matured DCs (TLDCs or SPDCs or unprimed DCs) were incubated with allogenic non adherent PBMCs at a 1:50 ratio for 24 h at 37 °C /5% CO_2_ in IFNγ captured antibody coated wells. The lymphocyte enriched autologous PBMCs were also cultured similarly with matured DCs at 1:10 ratio for 2 weeks and incubated for 48 h in IFNγ captured antibody coated wells in the presence of 100 IU/ml IL2 along with respective matured DCs*.* To avoid inter sample variation we multiplied the spot count with the spot size which is represented as the IFNγ spot Index for each patient. Following manufacturer's instructions spots were scanned using Immunospot versa analyser (CTL, Shaker Heights, OH, USA) using ImmunoCapture software version 6.4. The spots were then counted using the ImmunoSpot 5.0 ProDC software.

### Cytokines analysis by ELISA

Patient (P7–P9) monocytes derived DCs either primed with rhSPAG9 or tumor lysates (P7–P9) were generated in 7 days. Post 7 days, SPDCs and TLDCs were cultured in plain Cell Gro medium for 24 h without the proinflammatory cytokine. All viable floating matured DCs were collected for DCs-PBMCs co-cultures experiments and supernatant (WO) was stored at − 80 °C for conducting the ELISA experiments. Subsequently, autologous DCs thus generated were further co-cultured with PBMCs for 7 days. Supernatants from each well were collected on Day 7 and stored at − 80 °C for conducting ELISA experiments. Both IL12p70 and p40 were quantitated separately using the respective ELISA kits (IL12p70 and IL12p40 kit; Biolegend, San Diego, CA, USA) according to the manufacturer's instructions. The color developed was read at 450 nm using Multiskan Ascent (Thermo Electron Corporation, Grand Island, NY, USA).

### Effect of Cisplatin treatment on autologous PBMC co-cultures with DCs

To examine the effect of Cisplatin (Kemoplat, Fresenius Kabi, Germany) on DCs function, three different concentrations 150 µM (Dose 1), 200 µM (Dose 2) and 400 µM (Dose 3) (where Dose 2 is equivalent to the clinical dosage) were used. SPDCs were seeded (1 ×  10^5^ cells) into 96 well plate and cultured for 24 h at 37 ℃ followed by three different doses of cisplatin treatment. Treated SPDCs were co-cultured with autologous PBMCs in 1:10 ratio (n = 3; patient number P10–12). The frequency of treatment was day 0, 3 and 5 for monitoring CFSE based proliferation, for up to eight generations which is usually attained within a week. DCs alone as control were treated for 48 h with cisplatin and phenotypic marker expression and migratory capacity were analyzed. All experiments were carried out in three independent patients (P10–P12).

### Cytotoxicity assay by live/dead staining

LIVE/DEAD Fixable Violet Dead Cell Staining dye was used to discriminate the live or dead cells as per recommended protocol of the manufacturer (Invitrogen, OR, USA). Briefly PBMCs were centrifuged and re-suspended in 100 µl of blocking buffer. Subsequently, the cells were stained with fixable live/dead stain (1 µg/ml) for 15 min. The cells were washed twice with PBS and fixed with 2% PFA for further analysis.

### CCR7 antibody staining

Matured SPDCs viability was confirmed by trypan blue staining. To study the expression of CCR7, the SPDCs (without drug treatment) were centrifuged washed once with PBS and suspended in 100 ul of blocking buffer and kept at room temperature for 15 min. Subsequently, cells were probed with anti- CCR7 monoclonal antibody (Abcam, UK) for 20 min. at room temperature. After incubation, the cells were washed with PBS to remove the unbound antibody and incubated further with the secondary antibody conjugated with AF405 (Themofisher Scientific, USA) for 30 min. at room temperature. Finally, cells were washed and suspended in 2% PFA for flow analysis.

### Statistical analysis

Mann–Whitney U test was done to compare unprimed DCs with SPDCs and TLDCs with SPDCs. One-way ANOVA (Analysis of variance) was used to compare the phenotypic and functional characteristics of untreated and cultures treated with three different doses of CDDP. All the analyses were done using GraphPad prism 7 software (GraphPad Software, Inc. USA).

## Results

### SPAG9 protein expression in cervical cancer tissue

**Fig. 1:**
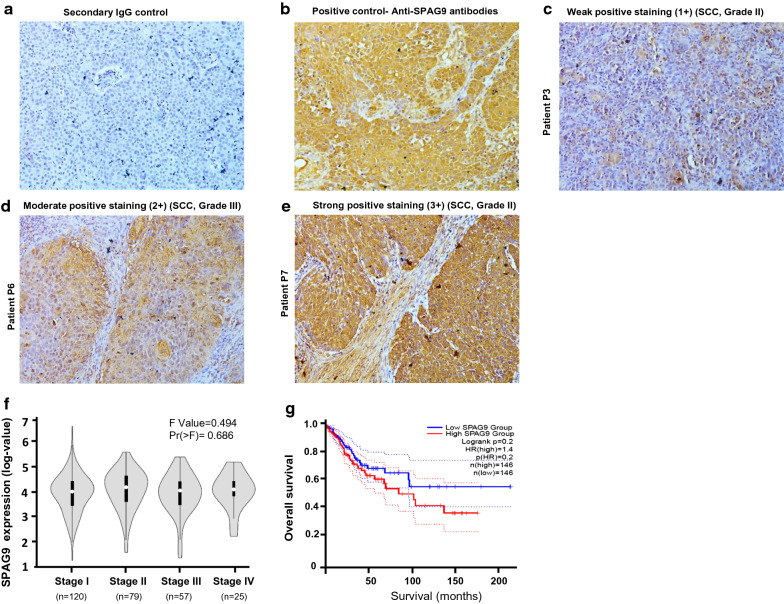
Immunohistochemical analysis of SPAG9 expression in cervical cancer tissues (**a**) Negative control (stained with secondary antibody only) (**b**) positive control probed with anti-SPAG9 antibody (**c**) weak positive staining (intensity 1+) (**d**) moderate (intensity 2+) (**e**) strong positive staining (intensity 3+) probed with anti-SPAG9 antibody. **f** Violin plot shows the expression of SPAG9 in different stages of TCGA-cervical cancer (CESC) patients datasets (n = 281), (**g**) Kaplan–Meier plot represents the overall survival of patients with low (blue) and high (red) expression of SPAG9 (vertical line represents event occurred) n = 146 in each category)

SPAG9 protein expression was probed in 12 cervical cancer patients using IHC (Table 1). Our histopathological analysis showed that two patients were found to have poorly differentiated carcinoma with SPAG9 protein expression (Table 1). The other 10 patients diagnosed with squamous cell carcinoma grade II or grade III (Table 1) also expressed SPAG9 but with varying intensity. SPAG9 immunoreactivity staining was graded on the following scale: 0, no staining; 1+ , weak staining; 2+ , moderate staining; 3+ , intense staining (Fig. 1a–e). Our results indicated that SPAG9 protein was expressed in all the patients irrespective of histotypes. Stage wise distribution of SPAG9 was analyzed in 281 samples of cervical squamous cell carcinoma and endocervical adenocarcinoma (CESC) from the cancer genome atlas data (TCGA; Fig. 1f). Further we evaluated SPAG9 association (low expression versus high expression) with overall survival of CESC (n = 146 in each group) TCGA dataset by Kaplan–Meier curve and compared by log-rank tests analysis (Fig. 1g). Our data revealed that CESC patients with high SPAG9 expression had poor prognosis with reduced survival compared to patients with low SPAG9 expression.

**Table 1:** The clinicopathological data along with the SPAG9 positivity and intensity scores by Immunohistochemistry

Patient ID	Age	Histopathology	SPAG9 positive tumor cells (%)	Intensity of SPAG9 expression
P1	54	SCC, Grade III	90	2–3+
P2	64	Poorly differentiated carcinoma	30	2–3+
P 3	45	SCC, Grade II	70	1–2+
P 4	48	SCC, Grade III	70	2+
P 5	56	SCC, Grade III	90	2+
P 6	45	SCC, Grade III	60	2+
P 7	49	SCC, Grade II	80	3+
P 8	54	Poorly differentiated carcinoma	80	3+
P 9	60	SCC, Grade III	90	2+
P 10	47	SCC, Grade III	80	2+
P 11	55	SCC, Grade III	80	2+
P 12	50	SCC, Grade III	40	2+

### Phenotypic characterization of SPAG9 primed matured DCs

**Fig. 2:**
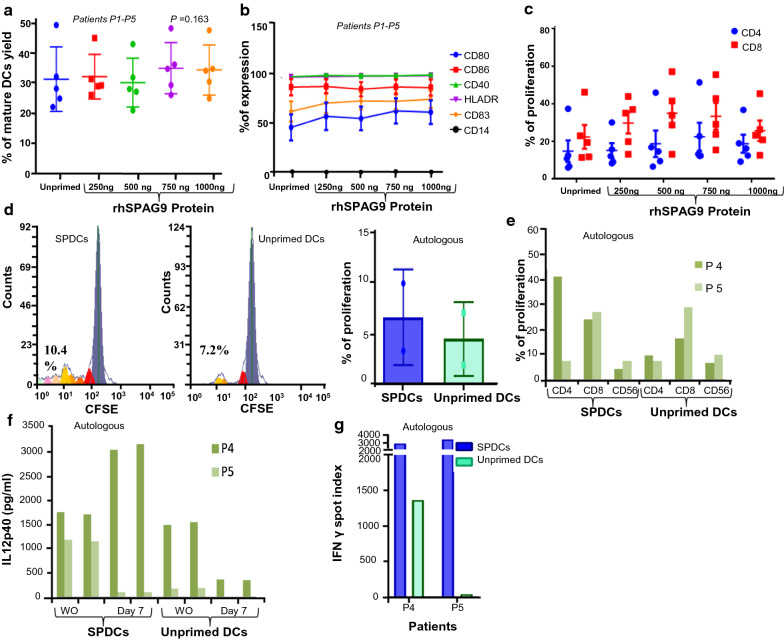
Optimization of rhSPAG9 antigen concentration for priming DCs (**a**) Percentage of mature DCs yield at different concentrations of rhSPAG9 priming. Comparative analysis of unprimed DCs and different concentration of rhSPAG9 primed DCs (**b**) Phenotypic characterization (**c**) Proliferation of allogenic CD4^+^ and CD8^+^ Tcells (PBMCs from N1**–**N5 healthy controls) (**d**) Overall proliferation of autologous PBMCsin response to SPDCs and unprimed DCs (P4–P5) (**e**) Proliferation of autologous CD4^+^and CD8^+^ Tcellsupon stimulation with SPDCs and unprimed DCs (**f**) Secretion level of IL12p40 in wash out (WO) and co-cultures supernatants of SPDCs and unprimed DCs (**g**) Secretion level of IFNγ by autologous PBMCsco-cultured withSPDCs and unprimed DCs (P4–P5). For Figs. 2(a–c), data represents summary of five different experiments (P1–P5)

Initially, we examined the effect of rhSPAG9 primed DCs at four different concentrations (250, 500, 750 and 1000 ng/ml) to study the phenotypic characterization in five patients (P1–P5). The percentage of live DCs primed with rhSPAG9 after 72 h was calculated. The highest yield of live DCs in culture media was observed with rhSPAG9 as compared to unprimed DCs and other concentrations of rhSPAG9 (Fig. 2a). Our data suggests that rhSPAG9 does not affect the phenotypic characterization of DCs as depicted in Fig. 2b. The expression of CD14 was down regulated in mature DCs irrespective of antigen priming. We further observed no difference in the expression of phenotypic markers between SPAG9 primed and unprimed DCs. However, when we performed an analysis within DCs primed with different concentrations of rhSPAG9, we found that, CD80 expression alone was slightly albeit insignificantly increased when 750 ng and 1000 ng/ml of rhSPAG9 (Fig. 2b). Additional file 1: Figure S1 shows the overlay of CD markers expressed in unprimed DCs and DCs primed with different concentration of rhSPAG9. This data suggests that maturation of DCs proliferation was not altered with rhSPAG9 pulsing.

### Optimization of rhSPAG9 concentration for DCs priming based on proliferation of allogeneic PBMCs

Using five patient samples (P1–P5), DCs were primed with various concentrations of rhSPAG9 (250, 500, 750 and 1000 ng/ml). Our results revealed that DCs primed with rhSPAG9 at a concentration of 500 ng and 750 ng/ml were having similar ability to induce proliferation of allogeneic PBMCs (N1–N5) compared to those primed either with rhSPAG9 250, 1000 ng/ml or with unprimed DCs. Further, we analyzed the status of CD4^+^ and CD8^+^ T cells in the dividing population. We found that CD8^+^ proliferation was significantly higher in 500 and 750 ng rhSPAG9 primed DCs (*P* = 0.006) compared to unprimed DCs. Also, CD4^+^ T cells response was higher in DCs primed with 750 ng of rhSPAG9 (Fig. 2c). Hence, we carried out all in vitro studies using 750 ng concentration of rhSPAG9 protein to prime DCs.

In two patients (P4 and P5) who had sufficient DCs for all the assays, we extended the comparison between 750 ng/ml rhSPAG9 primed and unprimed DCs to autologous PBMCs. We found that SPDCs were superior to unprimed DCs in stimulating proliferation. In P4, both CD4^+^ and CD8^+^ cells had proliferated (Fig. 2d–e) in response to SPDCs (CD8^+^/CD4^+^ ratio0.6) while in P5, a superior CD8^+^ T cell proliferation was seen (CD8^+^/CD4^+^ ratio3.6). However, in both patients, IL12p40 levels in washout cultures (Fig. 2f) as well as the IFN-γ response were higher in SPDCs co-cultures (Fig. 2g) as compared to unprimed DCs. However due to insufficient cell numbers, and poor unprimed DC yields, we could not compare them in a greater number of patients.

### Generation of TLDCs for comparison of efficacy

**Fig. 3:**
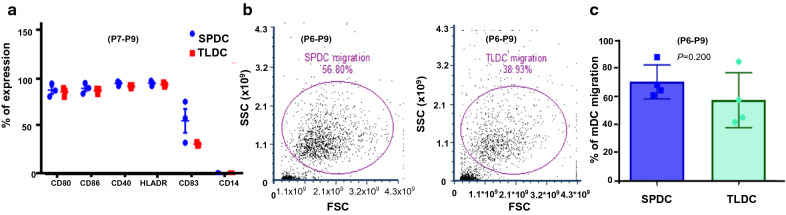
Characterization of mature DCs (**a**) Phenotypic characteristics of SPDCs and TLDCs (**b**) Dot plots representing the migratory ability of SPDCs and TLDCs towards the chemokinesCCL19 and CCL21 (**c**) Box and whisker plot shows data on the migratory capacity of SPDCs and TLDCs as a summary of four independent experiments (*P* = 0.200; P6–P9)

Tumor lysates pulsed DCs were generated from patients P6-P9 to compare with SPDCs. Our studies had previously established TLDCs as potent inducers of Th1 responses [25, 27]. However, tumor cells yield to pulse DCs at a 3:1 (tumor cell: DC) ratio is a serious limitation due to which, this study aims to evaluate rhSPAG9 as a reliable alternate. We divided immature DCs from the same patient (P7–P9) into two groups and pulsed one group with tumor lysates and the other group with rhSPAG9. When we compared the phenotypic expression of SPDCs and TLDCs, we did not find any significant difference in the expression of CD80, CD86, CD40 and HLA-DR but the maturation marker CD83 alone had an insignificant increase in SPDCs compared to TLDCs (*P* = 0.4; Fig. 3a).

### Migratory response of mature DCs toward CCL19 and CCL21

These experiments were performed in four independent patient samples (P6–P9). Interestingly, our results showed the migratory response of SPDCs to be slightly higher (70 ± 12%) as compared to TLDCs (57 ± 19.5%; P6–P9) although this was not significant (*P* = 0.2), indicating that SPDCs were capable of up regulating CCR7 in response to the chemokine cocktail CCL19 and CCL21 (Fig. 3b–c). We also found increased expression of CCR 7 in SPDCs of four patients (P 7 to 10) as shown in Additional file 1: Figure S2a–b.

### Induction of proliferation response by SPDCs

**Fig. 4:**
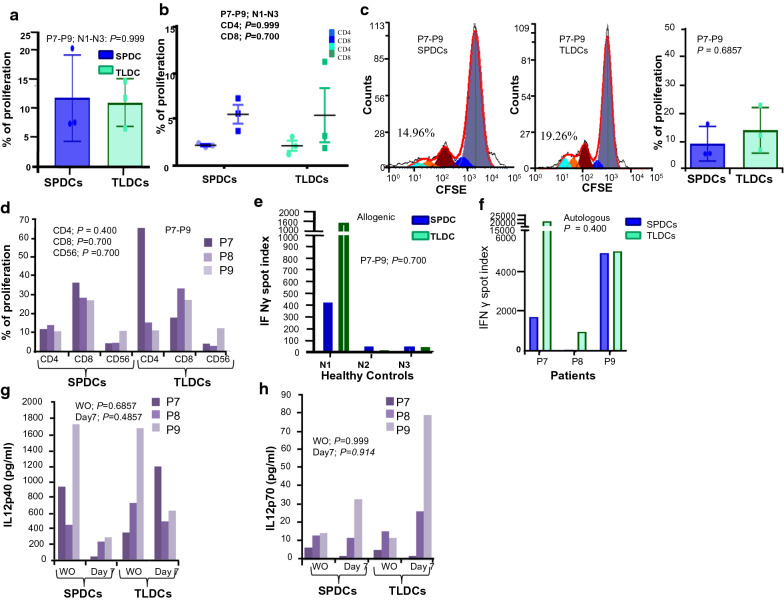
Functional characterization of mature DCs in allogenic and autologous co-cultures (**a**) Overall proliferation of allogenic PBMCs induced by SPDCs and TLDCs (*P* = 0.999) (**b**) Proliferation of allogenic CD4^+^ and CD8^+^ T cells in co-culture with DCs (N1-N3) (**c**) Representative histogram of overall proliferation of autologous PBMCs induced by SPDCs and TLDCs (**d**) Proliferation of autologous CD4^+^, CD8^+^ T cells and NK cells in response to SPDCs and TLDCs(CD4^+^; *P* = 0.400, CD8^+^; *P* = 0.700, CD56^+^; *P* = 0.700). **e** Secretion of IFNγ by allogenicPBMCs co-cultured supernatants of SPDCs and TLDCs (N1-N3: *P* = 0.700) (**f**) Secretion of IFNγ by autologous PBMCs co-cultured supernatants of SPDCs and TLDCs (P7-P9; *P* = 0.400) (**g**) The level of IL12p40in autologous wash out andco-cultured supernatants of SPDCs and TLDCs(WO—Wash out; *P* = 0.685 and at day7; *P* = 0.485) (**h**) The level of IL12p70 (WO—Wash out; *P* = 0.999 and at day7; *P* = 0.914) in wash out andautologous co-cultured supernatants of SPDCs and TLDCs. All individual experiments were done in duplicates and data shown from all autologous experiments is from three different patients for the SPDCsand TLDCs experiments (P7–P9)

We further compared the functional efficacy of both SPDCs and TLDCs to induce allogenic (N1–N3) as well as autologous PBMCs (P7–P9) to proliferate, using DCs generated from P7–P9 patients. Our data revealed no significant difference in the ability of SPDCs or TLDCs to induce proliferation of allogenic responders (Fig. 4a). The number of CD4^+^ T and CD8^+^ T cells was also similar in both allogenic co-cultures (Fig. 4b) indicating that both SPDCs and TLDCs were capable of stimulating the responders with similar efficacy to proliferate. Next, we examined proliferation response with autologous PBMCs co-cultures of SPDCs and TLDCs in the same patients (P7–P9). A representative histogram of proliferating cells induced by SPDCs and TLDCs is shown in Fig. 4c. The mean percentage of overall proliferation in SPDCs was 10.34% (± 6.4) as compared to 13.56 (± 10.8) in TLDCs co-cultures (Fig. 4c). Further, we found a similar NK cells (CD56^+^) response in both co-cultures; primed with SPDCs or TLDCs; CD4^+^ and CD8^+^ Tcell proliferation were similar in two patients (P8 and P9). However, in patient P7, CD8^+^T cells showed higher proliferation with SPDCs stimulation whereas CD4^+^T cell proliferation was increased when stimulated with TLDCs (Fig. 4d). SPAG9 is as good as tumor lysates and can be used for DCs pulsing for further experiments or clinical trials.

We subsequently compared the ability of the differently primed DCs to induce a Th1 response. In one patient (P6) due to less dendritic cell yield we were unable to perform the proliferation, IL12 ELISA and IFNγ ELIspot assays.

### IFN-gamma response in SPAG9 DC co-cultures

In order to compare the IFN-**γ** response, SPDCs or TLDCs were co-cultured with allogenic (N1–N3) and autologous PBMCs (P7–P9). SPDCs and TLDCs prepared from patient P7 were co-cultured with allogenic PBMCs isolated from normal healthy donor (N1). Similarly, SPDCs and TLDCs prepared from patients (P8 and P9) were co-cultured with allogenic PBMCs isolated from normal healthy donors (N2 and N3) respectively. Our data revealed that the spot index of IFNγ was similar in SPDCs and TLDCs co-cultures of patients P8 and P9, (Fig. 4e). However, in allogenic PBMC from N1 alone, we observed that TLDCs induced cultures had a four-fold difference compared to SPDCs induced cultures. The same trend was observed in autologous cultures also where we observed that TLDCs co-cultures from patient (P7) had the highest spot index compared to SPDCs (Fig. 4f). Upon further analysis, we also found that proliferation of CD4^+^ cells of P7 was also very high (> fourfold) in autologous TLDCs co-cultures as compared to those stimulated by SPDCs (Fig. 4d).

### IL12p40 and IL12p70 secretory response of SPDCs and TLDCs

In order to compare the Th1 response in autologous PBMCs (P7–P9) co-cultured with SPDCs and TLDCs, we evaluated the secretion of IL12 in cytokine withdrawn wash out (WO) supernatants after 24 h. We observed that the secretion of IL12p40 was higher in the SPDCs- WO as compared to TLDCs-WO supernatants (Fig. 4g). Similarly, the co-culture supernatants of TLDCs at day 7 showed higher levels of IL12p40 secretion when compared with SPDCs. In contrast, the secretion of IL12p70 was minimal in the WO supernatants of both TLDCs and SPDCs. (Fig. 4h). When we compared the ratio of IL12p40/p70 in co-culture supernatants, it was 19.5 for SPDCs and 21.5 for TLDCs in one patient (P8) and 8.5 and 7.8 respectively for another (P9) which indicated that the levels differed very little, within the same patient’s supernatants. However, in the third patient (P7), IL12p40 /p70 secretion was higher in TLDCs (595.7) compared to the SPDCs. It is noteworthy that IFN gamma secretion was the highest in both allogenic and autologous TLDC co-cultures of the same patient.

Immuno-histochemical analysis showed that all the three patients (P7–P9) had similar levels of SPAG9 expression in their tumor (80–90%) with 2 + intensity (Table 1), leading us to speculate that tumor lysates could have a varying mixture of tumor associated antigens with differing antigenicity leading to variation in response levels compared to the single protein antigen rhSPAG9.

### Evaluating the cytotoxic effect of cisplatin on SPDCs

**Fig. 5:**
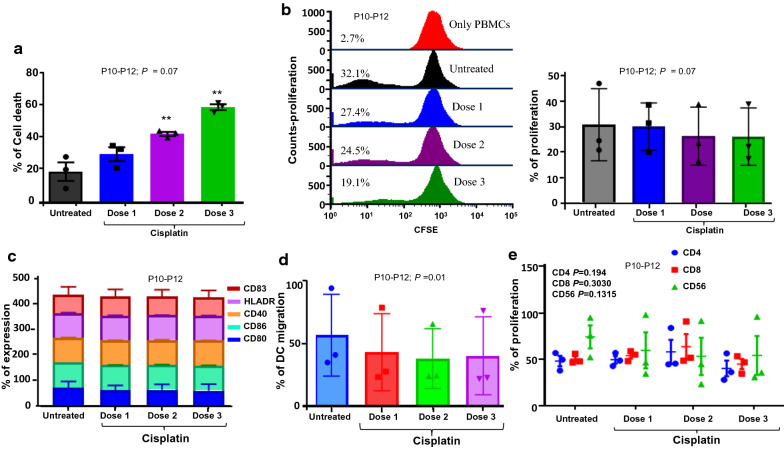
Characterization of SPDCs in the presence of chemotherapeutic agent cisplatin at three different doses (150, 200 and 400 µM/ml) (**a**) Bar diagram depicting cell death post cisplatin treatment (**b**) Overall proliferation of autologous PBMCs SPDCs in the presence or absence of cisplatin. **c** Phenotypic characterization of SPDCs in the presence or absence of cisplatin (**d**) Evaluation of migratory capacity of SPDCs with or without the cisplatin in culture medium (*P* = 0.01) (**e**) Overall proliferation of CD4^+^ (*P* = 0.194), CD8^+^ (*P* = 0.303) and NK cell response (*P* = 0.131) to SPDCs (± Cisplatin). Data shown is summary of experiments with three different patients (P10–P12)

We further examined the effect of cisplatin on SPDCs phenotype, viability, and their migratory capacity in P10-P12 patients. Our results indicated that there was significant cell death in dose 2 (200 µM; *P* = 0.034) and dose 3 (400 µM; *P* = 0.032) cisplatin treated SPDCs as compared to untreated controls (Fig. 5a). We also, analyzed the proliferation of autologous PBMCs upon SPDCs stimulation in the presence or absence of cisplatin. The overall proliferation in dose 2 and dose 3 of cisplatin treated SPDCs culture was insignificantly decreased compared to the dose 1 (150 µM) and untreated cultures (Fig. 5b). Further, the co-stimulatory or maturation markers were not significantly affected in any of the cisplatin treated SPDCs compared to untreated controls (Fig. 5c). In addition, we found an insignificant decrease in CD80 and increase in HLADR expression upon cisplatin treatment (Fig. 5c). However, we observed a significant decrease in the migration of SPDCs at 400 µM concentration alone (*P* = 0.01; Fig. 5d). This showed that after 48 h of cisplatin treatment, most of the SPDCs retained their viability, phenotypic and migratory characteristics.

**Fig. 6:**
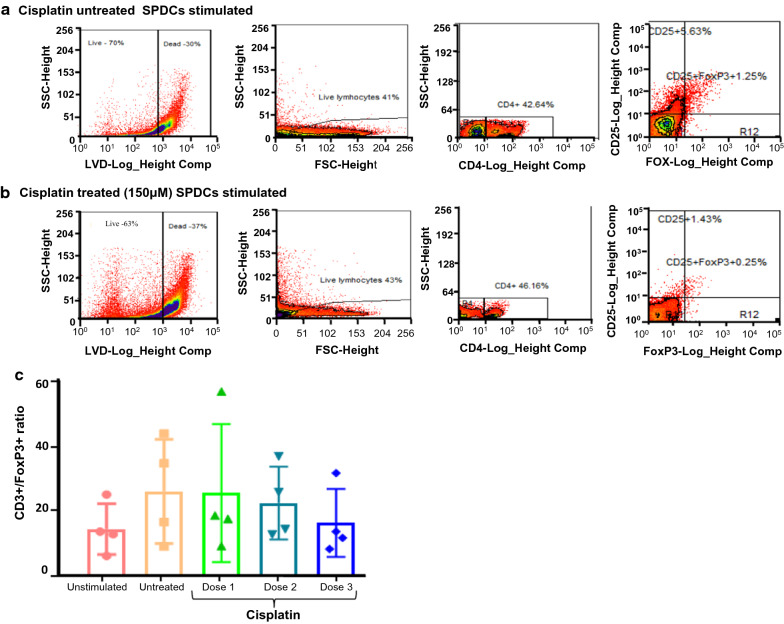
FACS analysis of CD3^+^/FoxP3^+^ Tregs in (**a**) Cisplatin untreated SPDCs stimulated co-cultures (**b**) Cisplatin treated (150 μM) SPDCs stimulated co-cultures (**c**) Bar graphs indicate CD3^+^/ FoxP3^+^Tregs ratio in SPDCs stimulated co-cultures treated with three different doses of cisplatin treated SPDCs (*P* = 0.340; P10–P12)

We further observed a significant decrease in Tcells and NK cell (CD56^+^) compartment with highest dose (400 µM) of cisplatin treated cultures alone (*P* = 0.02; Fig. 5e). Interestingly, we observed an increased proliferation of CD4^+^ and CD8^+^ T cells in dose 2 treated co-cultures (Fig. 5e). We further evaluated if an increase in the FOXP3^+^ compartment led to the rise in proliferation. We calculated the ratio of total CD3 + /FOXP3 + T-cell population and found a slight decrease in the ratio only in dose 3 treated co-cultures. However, we speculate the decrease in the ratio could be due to an overall increase in the death of proliferating T cells at the highest dosage (Fig. 6a–c, Additional file 1: Figure S2c) indicating that Tregs did not undergo proliferation and hence were not affected by the highest dose of cisplatin.

## Discussion

Cervical cancer is one of the major gynecological cancers leading to cancer related deaths worldwide [1]. In India, majority of cervical cancers (85%) are diagnosed at advanced stages and more than half (63%–89%) have regional disease at the time of presentation [29]. Recently, SPAG9 was shown to be associated with cervical cancer [17] which also showed that majority of the patients generated humoral response against SPAG9 protein suggesting that SPAG9 is a highly immunogenic protein. These findings are thus suggestive of SPAG9 as potential target for the development of novel therapeutic interventions. In the current study, our results confirmed the expression of SPAG9 protein] in all histotypes and grades of cervical cancer patients who were included for this investigation. The goals of cancer immunotherapy are to activate and expand tumor-specific CD4^+^ and CD8^+^ T cells as effective means of augmenting immunity to reduce tumor burden. Therefore, initially we examined the various concentrations of rhSPAG9 for generating SPDCs that showed effective Th1 polarization and found that priming DCs with 750 ng/ml of rhSPAG9 in the presence of proinflammatory cytokines was most effective. In addition, the generated SPDCs expressed both maturation and costimulatory markers and had an increased migratory capacity. We also observed that the SPDCs were capable of inducing a Th1 response both in allogenic and autologous responders by increasing the proliferation of CD8 + CTL compared to T helper cells. We also confirmed that cisplatin which is used as a standard chemotherapeutic agent for cervical cancer treatment, did not inhibit the efficacy of SPDCs. In addition, cisplatin at a concentration of 150 μM and 200 μM dosage did not impede the proliferation of CD4^+^ and CD8^+^ T cells significantly.

Priming of DCs with whole protein or whole tumor lysates from autologous tumor have been used in treating numerous cancers. The major advantages of this method compared to peptides are the multiple epitopes presented by MHC molecules that can activate both CD4^+^ and CD8^+^ T cells effectively towards a wide spectrum of antigens. Along with this, the requirement for processing of antigens results in prolonged antigen presentation [30]. The use of whole cancer-testis antigen proteins such as NY-ESO-1 and MAGE A3 even when given alone were found mediating both humoral and cellular immune response [31]. A recent phase I study conducted in advanced cancers showed that administration of LV305, a novel lentivirus based cancer vaccine that induces the expression of NY-ESO1 in DCs, resulted in potential clinical activity with the induction of antigen specific response in sarcoma, melanoma, ovarian and lung cancers [32]. Our current study shows that the priming of DCs with the novel rhSPAG9, showed comparable increase in maturation markers with statistically insignificant increased migratory potential in comparison to whole tumor lysate pulsed DCs. Some maturation cocktails contain PGE2 in order to improve migratory properties of DCs. We found SPDCs to be surprisingly more robust at migrating towards the CCR7 ligands CCL19 and 21, across a chemokine gradient even without PGE2 in the maturation cocktail. PGE2 is controversial for its ability to cause infiltration of suppressive cells expressing the enzyme Indoleamine 2,3, dioxygenase (IDO1) which in turn caused the infiltration of FOXP3 + regulatory cells [33].

SPDCs were capable of eliciting a potent Th1 response even at a concentration of 750 ng/ml. However, in comparison, studies have shown that peptides need to be used at a much higher concentration (up to 10 ug/ml) [34]. Several studies also reported that CTAs have restriction epitopes identified as the recognition sites for CD8^+^ T lymphocytes [35–37].

A clinical trial conducted in pancreatic cancer patients treated with a combinatorial treatment using the Onivyde and leucovorin /5FU regimen followed by 6 doses of autologous dendritic cell vaccine showed a substantial decrease in the tumor burden [38]. In addition, studies also showed a reduction in Treg/MDSC cells along with tumor specific CD4 + and CD8 + T cell generation in patients when dendritic cell vaccines were combined with chemotherapeutic regimen [39–41]. In this context, we studied the effect of cisplatin on SPAG9 primed DCs and the responding immune cells. Our study showed that cisplatin treatment did not downregulate phenotypic expression even at higher concentration. Further, the percentage of HLADR was slightly increased in cisplatin treated cultures compared to untreated cells which is in line with the results reported earlier [42], that showed an increase in the expression of HLA class II upon treatment of dendritic cells with paclitaxel [41]. In a different study, it was documented that addition of cisplatin during DC differentiation did not inhibit T cell proliferation [42]. Our study also shows that cisplatin treatment of the differentiated rhSPAG9 primed DCs did not inhibit their ability to stimulate helper and cytotoxic cells to proliferate.

Similarly, several studies [43–45] have shown the importance of the ratio between CD4+ /, CD8+ / or CD3+ and FOXP3+ cells as being crucial in predicting prognosis of cancer patients. Hence, we wanted to verify the effect of SPDC stimulation in-vitro on the CD3+ /FOXP3+ ratio in the presence or absence of cisplatin. However we did not find any significant decrease in the ratio at the clinical dose equivalent. This presents the possibility of combining vaccination using SPDCs with a cisplatin based chemotherapeutic regimen in the treatment of advanced cervical cancer.

## Limitation

This study was conducted in 12 cervical cancer patients with a limitation of blood volume (20 ml) collected because of the fact that patients had to undergo the process of blood collection for the routine clinical diagnosis in the hospital. Yet another limitation was that the PBMCs thus isolated from blood, were subjected to various assays in triplicate such as phenotype, migration, proliferation, Th1 response and co-culturing assays. Hence, the spectrum of response was variable due to variations in pathological features and immunological differences among the small cohort of patients (12 cervical cancer patients). Further studies are warranted in large number of patients that will be done in our ongoing Phase II cervical cancer trials.

## Conclusion

In conclusion, this preclinical report provides evidence of a potent Th1 response in vitro, produced by the novel rhSPAG9 protein primed DCs similar to that of TLDCs, and may be combined with the chemotherapeutic drug cisplatin for the treatment of cervical cancer patients in advanced stages of the disease.

## Supplementary Information

**Additional file 1: Figure S1.** Representative FACS profile of CD markers expression in unprimed and primed DCs at various concentrations of SPAG9 (**a**) CD80 (**b**) CD86 (**c**) CD14 (**d**) CD83 (**e**) CD40 and (**f**) HLADR. **Figure S2.** (**a**) Representative FACS profile for CCR7 expression in SPDCs (**b**) Bar graph indicates the CCR7 expression in four patients (P7–P10) (**c**) Fluorescence Minus One (FMO) control for gating the FoxP3 + population.

## Data Availability

Not applicable.

## Abbreviations

ANOVA: Analysis of variance
APC: Allophycocyanin
CD: Cluster of differentiation
CDDP: Cis-di amine di chloride platinum (II)
DCs: Dendritic cells
ECD: Energy Coupled Dye
ELISA: Enzyme-Linked Immunosorbent assay
ELISpot: Enzyme-Linked Immune absorbent spot
FITC: Fluorescein isothiocyanate
FOXP3: Fork head box P3
GMCSF: Granulocyte–Macrophage colony stimulation factor
HPV: Human papilloma virus
IFN: Interferon
IL: Interleukin
MHC: Major Histocompatibility complex
MIP: Macrophage inflammatory protein
NK: Natural killer
PBMCs: Peripheral blood mononuclear cells
PC5: Phycoerythrin Cyanine 5
PC7: Phycoerythrin Cyanine 7
PE: Phycoerythrin
SPDC: SPAG9 primed DCs
TLDCs: Tumor Lysate Dendritic Cells
TLR: Toll like receptor
TNF: Tumor necrosis factor
Tregs: T regulatory cells
TS: Type specific
WIA: Women’s India Association
WO: Wash out
