# Supplementary material for: Dendritic cells matured with recombinant human sperm associated antigen 9 (rhSPAG9) induce CD4+, CD8+ T cells and activate NK cells: a potential candidate molecule for immunotherapy in cervical cancer
Source: Cancer Cell Int. 2021 Sep 7;21:473. doi: 10.1186/s12935-021-01951-7 (PMC8424976; doi:10.1186/s12935-021-01951-7)
Supplement: Supplementary file 1 — Additional file 1: Figure S1. Representative FACS profile of CD markers expression in unprimed and primed DCs at various concentrations of SPAG9 (a) CD80 (b) CD86 (c) CD14 (d) CD83 (e) CD40 and (f) HLADR. Figure S2. (a) Representative FACS profile for CCR7 expression in SPDCs (b) Bar graph indicates the CCR7 expression in four patients (P7–P10) (c) Fluorescence Minus One (FMO) control for gating the FoxP3 + population. [file 12935_2021_1951_MOESM1_ESM.pptx]

## Slide 1
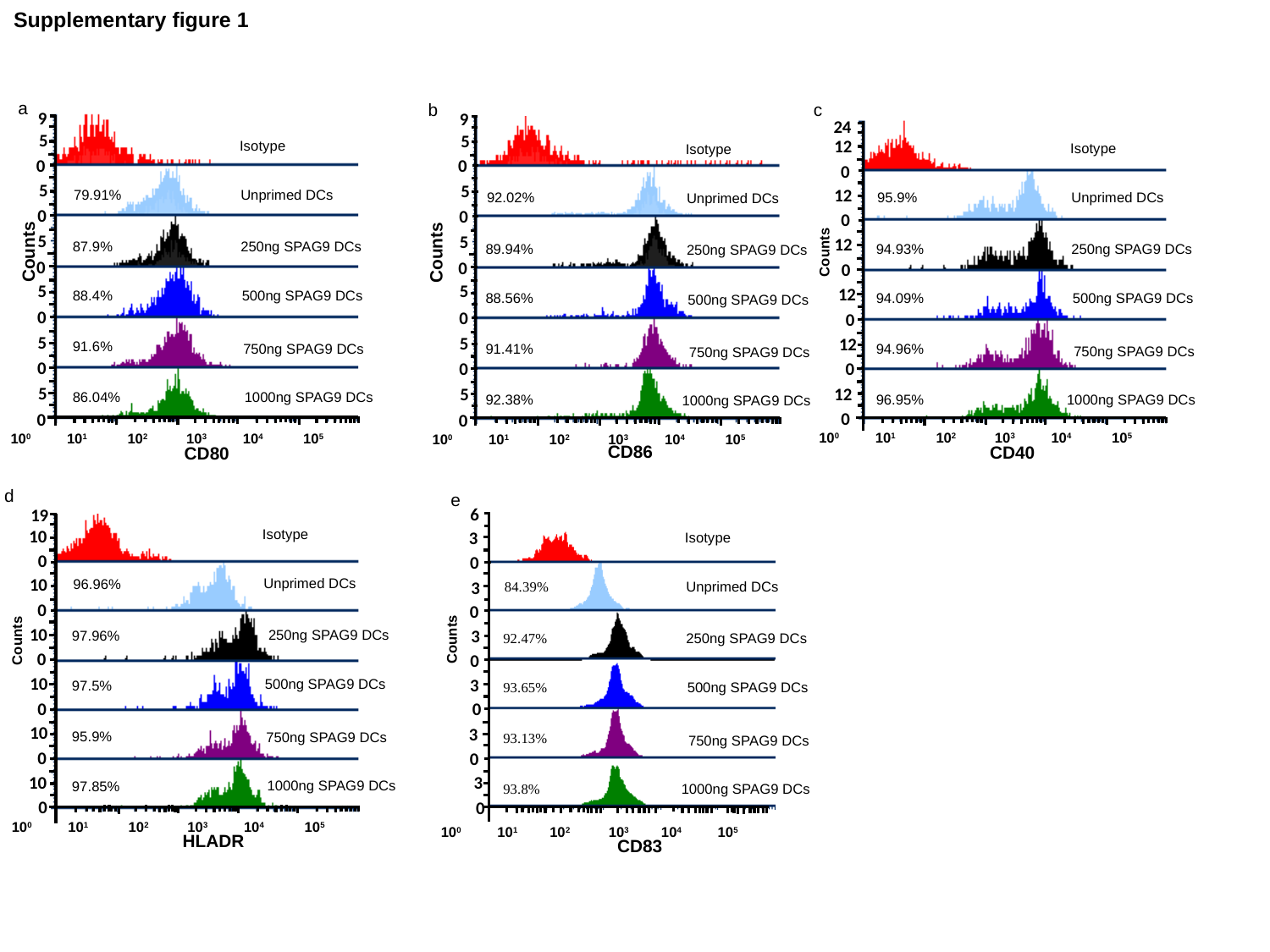

Supplementary figure 1
a
b
c
24
12
0
12
0
12
Counts
0
12
0
12
0
12
0
100 101 102 103 104 105
CD40
Isotype
95.9%
Unprimed DCs
250ng SPAG9 DCs
94.93%
500ng SPAG9 DCs
94.09%
94.96%
750ng SPAG9 DCs
1000ng SPAG9 DCs
96.95%
9
5
0
5
0
5
Counts
0
5
0
5
0
5
0
100 101 102 103 104 105
9
5
0
5
0
5
Counts
0
5
0
5
0
5
0
100 101 102 103 104 105
CD86
Isotype
Unprimed DCs
250ng SPAG9 DCs
500ng SPAG9 DCs
750ng SPAG9 DCs
1000ng SPAG9 DCs
79.91%
87.9%
88.4%
91.6%
86.04%
CD80
Isotype
92.02%
Unprimed DCs
89.94%
250ng SPAG9 DCs
88.56%
500ng SPAG9 DCs
91.41%
750ng SPAG9 DCs
92.38%
1000ng SPAG9 DCs
d
e
6
3
0
3
0
3
Counts
0
3
0
3
0
3
0
100 101 102 103 104 105
CD83
19
10
0
10
0
10
Counts
0
10
0
10
0
10
0
100 101 102 103 104 105
HLADR
Isotype
Unprimed DCs
96.96%
250ng SPAG9 DCs
97.96%
500ng SPAG9 DCs
97.5%
95.9%
750ng SPAG9 DCs
1000ng SPAG9 DCs
97.85%
Isotype
Unprimed DCs
250ng SPAG9 DCs
500ng SPAG9 DCs
750ng SPAG9 DCs
1000ng SPAG9 DCs
84.39%
92.47%
93.65%
93.13%
93.8%

## Slide 2
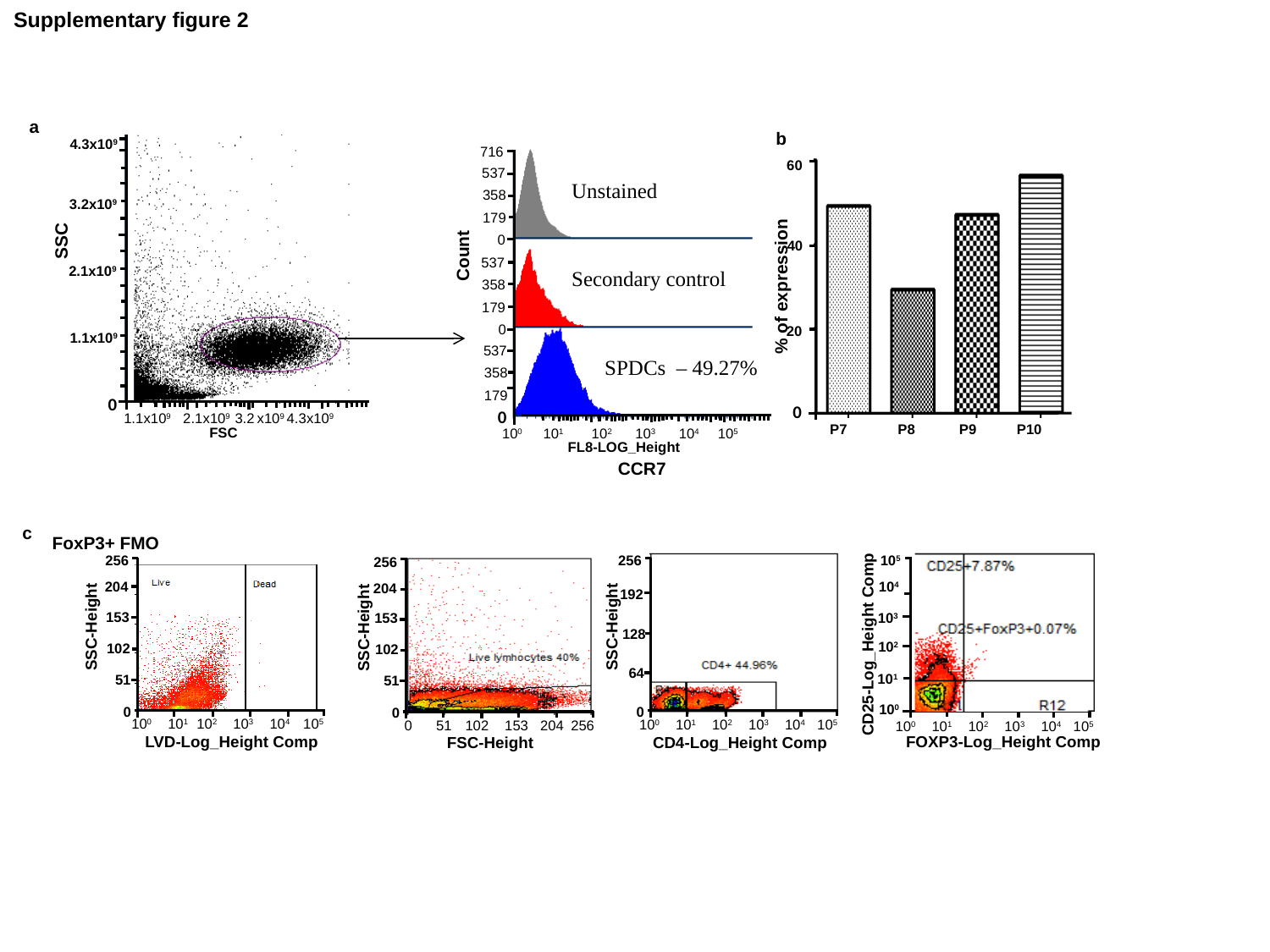

Supplementary figure 2
a
b
4.3x10^9^
SSC
0
1.1x10^9^ 2.1x10^9^ 3.2 x10^9^ 4.3x10^9^
3.2x10^9^
2.1x10^9^
1.1x10^9^
FSC
716
60
40
% of expression
20
0
P7	 P8 P9 P10
537
Unstained
Secondary control
SPDCs – 49.27%
358
179
0
Count
537
358
179
0
537
358
179
0
100 101 102 103 104 105
FL8-LOG_Height
CCR7
c
FoxP3+ FMO
CD25-Log_Height Comp
102
101
100
100 101 102 103 104 105
FOXP3-Log_Height Comp
105
104
103
256
204
153
SSC-Height
102
51
0
 100 101 102 103 104 105
LVD-Log_Height Comp
256
192
SSC-Height
128
64
0
100 101 102 103 104 105
CD4-Log_Height Comp
256
204
153
SSC-Height
102
51
0
0 51 102 153 204 256
FSC-Height
